# An efficient Dai-Yuan projection-based method with application in signal recovery

**DOI:** 10.1371/journal.pone.0300547

**Published:** 2024-06-10

**Authors:** Jamilu Sabi’u, Ado Balili, Homan Emadifar

**Affiliations:** 1 Department of Mathematics, Faculty of Science, Yusuf Maitama Sule University, Kano, Nigeria; 2 Department of Mathematics, Saveetha School of Engineering, Saveetha Institute of Medical and Technical Sciences, Saveetha University, Chennai, Tamil Nadu, India; 3 MEU Research Unit, Middle East University, Amman, Jordan; 4 Department of Mathematics, Hamedan Branch, Islamic Azad University, Hamedan, Iran; Michigan State University, UNITED STATES

## Abstract

The Dai and Yuan conjugate gradient (CG) method is one of the classical CG algorithms using the numerator ‖*g*_*k*+1_‖^2^. When the usual Wolfe line search is used, the algorithm is shown to satisfy the descent condition and to converge globally when the Lipschitz condition is assumed. Despite these two advantages, the Dai-Yuan algorithm performs poorly numerically due to the jamming problem. This work will present an efficient variant of the Dai-Yuan CG algorithm that solves a nonlinear constrained monotone system (NCMS) and resolves the aforementioned problems. Our variant algorithm, like the unmodified version, converges globally when the Lipschitz condition and sufficient descent requirements are satisfied, regardless of the line search method used. Numerical computations utilizing algorithms from the literature show that this variant algorithm is numerically robust. Finally, the variant algorithm is used to reconstruct sparse signals in compressed sensing (CS) problems.

## Introduction

Firstly, we consider the nonlinear minimization problem

$$\begin{array}{c} \underset{x\in R^{n}}{\text{min}}\;f\left(x\right), \end{array}$$ (1)

in which *f* is an *n*-variables smooth function from $R^{n}\to R$. The following notations will be employed throughout this work: ∇*f*(*x*_*k*_) = *g*(*x*_*k*_) = *g*_*k*_, and *f*(*x*_*k*+1_) = *f*_*k*+1_. It has become a trend in nonlinear optimization to solve (1) by employing the conjugate gradient (CG) method, which is an algorithm that requires minimum memory to implement. Given an arbitrary $x_{0}\in R^{n}$, the method takes the following form:

$$\begin{array}{c} x_{k+1}=x_{k}+s_{k},\;s_{k}=\vartheta_{k}d_{k},\;\;k=0,1,\ldots , \end{array}$$ (2)

where *x*_*k*_ stands for the *k*^*th*^ iterate, *ϑ*_*k*_ > 0 is a steplength that is often obtained by applying an appropriate line search procedure [1–6]. The CG direction *d*_*k*_ given by

$$\begin{array}{c} d_{0}=-g_{0},\;\;k=0,\;d_{k+1}=-g_{k+1}+\beta_{k}d_{k},\;\;k>0, \end{array}$$ (3)

in which *β*_*k*_ has a significant impact on the robustness of different CG algorithms. Over the decades, various formulations of the parameter have been proposed. The classical *β*_*k*_ updates are defined as follows:

$$\begin{array}{c} \beta_{k}^{FR}=\frac{∥g_{k+1}{∥}^{2}}{∥g_{k}{∥}^{2}}[7],\;\;\beta_{k}^{CD}=\frac{∥g_{k+1}{∥}^{2}}{-d_{k}^{T}g_{k}}[8],\;\;\beta_{k}^{DY}=\frac{∥g_{k+1}{∥}^{2}}{d_{k}^{T}y_{k}}[9], \end{array}$$ (4)

$$\begin{array}{c} \beta_{k}^{HS}=\frac{g_{k+1}^{T}y_{k}}{d_{k}^{T}y_{k}}[10],\;\;\beta_{k}^{PRP}=\frac{g_{k+1}^{T}y_{k}}{∥g_{k}{∥}^{2}}[11,12],\;\;\beta_{k}^{LS}=\frac{g_{k+1}^{T}y_{k}}{-d_{k}^{T}g_{k}}[13], \end{array}$$ (5)

in which ‖⋅‖ stands for the *ℓ*_2_ − *norm* of vectors. For more *β*_*k*_ updates, the reader should see [1, 2, 14, 15]. A descent condition (DC) with respect to a CG direction is satisfied if

$$\begin{array}{c} d_{k+1}^{T}g_{k+1}<0, \end{array}$$ (6)

and also satisfied the sufficient DC if

$$\begin{array}{c} d_{k+1}^{T}g_{k+1}\leq -c{∥g_{k+1}∥}^{2},\;\;c>0. \end{array}$$ (7)

In most circumstances, proving the sufficient DC (7) is enough to analyze global convergence for numerous CG algorithms.

Now, observe that the parameters in (4) and (5) have the common numerator ‖*g*_*k*+1_‖^2^ and $g_{k+1}^{T}y_{k}$ respectively. It was noted in [14] that the global convergence theorem of CG methods with *β*_*k*_ defined in (4) depends only on the Lipschitz condition and is independent of the boundedness assumption. Also, CG schemes with the update parameters in (4) converge globally to the minimizer of objective function *f* in (1) when the step-size *ϑ*_*k*_ is computed exactly, namely, *ϑ*_*k*_ is determined as

$$\begin{array}{c} \vartheta_{k}=\text{arg}\;\underset{\vartheta >0}{\text{min}\;}\;f\left(x_{k}+\vartheta d_{k}\right). \end{array}$$

Despite this, the performance of methods using parameters in (4) is not encouraging. This is because they are prone to jamming, which occurs frequently when the algorithms perform multiple steps that do not approach the minimizer. On the other hand, compared to the schemes in (4), the CG methods with the parameters in (5) exhibit nice numerical performance because they possess an automatic restarting mechanism that avoids the jamming phenomena experienced by the methods with *β*_*k*_ in (4). However, these methods converge globally only when *f* is a strongly convex function, namely,

$$\begin{array}{c} f\left(x\right)=b^{T}x+\frac{1}{2}x^{T}Ax,\;\;b\in R^{n}, \end{array}$$ (8)

where A is a positive-definite and symmetric matrix, the exact line search is employed and the step size approaches zero whenever the Lipschitz condition is assumed. Now, it is important to note that the methods with *β*_*k*_ defined by (4) and (5) are equivalent whenever *f* satisfies (8) and the exact line search is employed. The schemes also generate the pure *conjugacy* condition

$$\begin{array}{c} d_{i}^{T}Ad_{j}=0,\;\;\forall i\neq j, \end{array}$$ (9)

which ensures the minimization of *f* in at most *n* steps holds with A as defined earlier. Now, let *y*_*k*_ be as defined in (1). Then utilizing the mean-value theorem for general nonlinear functions, there exists *τ* ∈ (0, 1) such that

$$\begin{array}{c} d_{k+1}^{T}y_{k}=\alpha_{k}d_{k+1}^{T}\nabla^{2}f\left(x_{k}+\tau \alpha_{k}d_{k}\right)d_{k}, \end{array}$$ (10)

holds. Considering (9) and (10), it is ideal to consider the conjugacy condition

$$\begin{array}{c} d_{k+1}^{T}y_{k}=0, \end{array}$$ (11)

which, combined with (3) yields the $\beta_{k}^{HS}$ update parameter presented in (5). Furthermore, both conjugacy condition (9) and (11) are implemented only with the exact line search condition which is not feasible in practical computations. Following this development, Perry employed the quasi-Newton condition to develop a modification of (11), which is used with inexact line searches namely

$$\begin{array}{c} d_{k+1}^{T}y_{k}=-g_{k+1}^{T}s_{k}. \end{array}$$ (12)

As a consequence of (12), Dai and Liao [16] incorporated a parameter *t* ∈ (0, ∞) in (12) and presented another variant of (12) as

$$\begin{array}{c} d_{k+1}^{T}y_{k}=-tg_{k+1}^{T}s_{k}. \end{array}$$ (13)

As the Dai-Yuan(DY) method possesses shortcomings of the CG methods with *β*_*k*_ in (4), attempts have been made over the years by researchers to develop its modifications for solving (1) with better performance and global convergence theorem [17–20]. In line with this, Andrei [21] proposed a scaled DY-type method for solving (1) with sufficient descent and conjugacy conditions. Andrei [22] also proposed a hybrid method for solving (1), where the parameter *β*_*k*_ of the scheme is a convex combination of the DY and HS parameters. Based on ideas of the quasi-Newton method by Li and Fukushima [23], Wei et al. [24], Zhang et al. [25], and Zhang [26] proposed two DY-type algorithms for solving (1). One of the schemes converges globally for nonconvex functions, while the second method exhibits the good performance properties it inherits from the HS method. For other DY-type methods proposed in the literature, see [27–34] and the references therein.

It has become a trend for nonlinear real-life problems, which are often modelled as (1), to also be formulated as the convex-constrained nonlinear problem

$$\begin{array}{c} F\left(\bar{x}\right)=0,\;\;\bar{x}\in C, \end{array}$$ (14)

in which C is a nonempty, closed convex subset of $R^{n}$, and $F:R^{n}\to R^{n}$ is continuous and monotone, i.e., *F* satisfies the inequality

$$\begin{array}{c} {\left(F\left(x\right)-F\left(y\right)\right)}^{T}\left(x-y\right)\geq 0,\;\;\forall x,y\in C. \end{array}$$ (15)

Furthermore, due to the notion of optimality condition and nice properties of the CG methods for solving (1) and (14), i.e., when ∇*f* = *F*, is regarded as a gradient mapping of (1), different methods for solving (14) have been proposed in recent decades by scholars working on large-scale problems. Search directions of the methods are defined by

$$\begin{array}{c} d_{0}=-F_{0},\;\;d_{k+1}=-F_{k+1}+\beta_{k}d_{k},\;\;k=0,1\ldots , \end{array}$$

where *β*_*k*_ is determined by (4), (5) and their modifications. The problem expressed in (14) arises in applications such as the *ℓ*_1_ − *norm* regularization problems in CP [35–40], the equilibrium problems in [41, 42], etc.

In recent years, variants of DY algorithms [30] have been developed to solve the problem in (14). This includes the spectral DY projection approach by Liu and Li [43], where the algorithm was obtained by combining the classical DY parameter [9], the spectral gradient method [44], and the projection algorithm proposed in [45]. In this scheme however, the $C=R^{n}$. Moreover, without considering the system’s differentiability assumption, the authors were able to prove the global convergence of their method. Also, in [46], Liu and Li combined the multivariate spectral gradient method [47] with the DY scheme [9] to present a spectral DY-type method for solving problem (14). Motivated by the work in [43, 48], Liu and Feng [49] presented a modified DY-type method for solving problem (14). The scheme’s derivative-free structure makes it ideal for solving large-scale non-smooth problems. By employing the Lipschitz continuity assumption, the authors proved the global convergence of the new method. Inspired by the work in [49], Sani et al. [50] presented a DY-type method for solving (14), where the search direction of the scheme is obtained as a convex combination of the unmodified DY and CD parameters. The authors proved the global convergence of the scheme under mild assumptions. Only recently, Alhobaiti et al. [51] proposed two scaled DY-type algorithms for solving (14), where two different approaches were applied to compute the scaling parameter. The authors also showed that both methods satisfy (7) irrespective of the line search strategy used.

We outline the work’s aims as:

- To develop an efficient DY-type scheme for solving the problem (14), which not only inherits nice properties of the classical DY method but avoids its shortcomings.
- To present a DY-type algorithm that ensures sufficient DC.
- To develop a method with restart and scaling properties for accelerating the convergence.
- To demonstrate global convergence and analyse the proposed method’s convergence rate.
- To demonstrate the application of the method in sparse signal reconstruction.

The paper is organised as follows: The motivation and details of the proposed method are discussed in the next section. Section three contains the convergence results. Section four presents a detailed discussion of numerical results. part five gives the scheme’s applications, while part six draws conclusions.

## Motivation and details of the method

We begin this section by stating the Cauchy-Schwartz inequality that will occasionally be recalled in the derivation as well as convergence analysis of the proposed algorithm. Let *u* and *v* be vectors in an inner product space, then

$$\begin{array}{c} |\langle u,v\rangle |\leq ∥u∥∥v∥, \end{array}$$ (16)

where 〈*u*, *v*〉 stands for inner product between *u* and *v*.

As known theoretically, the sufficient DC (7) holds for all the classical CG methods when the objective function is convex quadratic and the exact line search procedure is employed. In actual computations, however, where inexact line searches are used, (7) does not hold in general for these methods. For example, by substituting the HS parameter in (3) and multiplying through by $g_{k+1}^{T}$, we obtain

$$\begin{array}{c} d_{k+1}^{T}g_{k+1}=-{∥g_{k+1}∥}^{2}+\beta_{k}^{HS}g_{k+1}^{T}d_{k}. \end{array}$$ (17)

Clearly, (17) satisfies the sufficient DC if $\beta_{k}^{HS}\geq 0$ and $g_{k+1}^{T}d_{k}\leq 0$. On the other hand, if $\beta_{k}^{HS}\geq 0$ and $g_{k+1}^{T}d_{k}>0$ the condition may not hold as the quantity $\beta_{k}^{HS}g_{k+1}^{T}d_{k}$ may become larger than −‖*g*_*k*+1_‖^2^. To remedy this defect of the HS method, Dong et al. [52] made some modifications to the method that not only satisfies the sufficient DC but also inherits the nice attributes of the scheme. The authors proposed the following modified HS search direction:

$$\begin{array}{c} d_{k+1}^{D}=\{\begin{array}{c} -\lambda_{k+1}g_{k+1}+\beta_{k}^{D}d_{k},\;g_{k+1}^{T}d_{k}>0,\;k\geq 0;\\ -g_{k+1}+\beta_{k}^{HS}d_{k},\;g_{k+1}^{T}d_{k}\leq 0,\;k\geq 0,\;\;\text{otherwise}. \end{array} \end{array}$$ (18)

In (18), λ_*k*+1_ is given by

$$\begin{array}{c} \lambda_{k+1}=1+\frac{g_{k+1}^{T}d_{k}}{d_{k}^{T}y_{k}}.\frac{g_{k+1}^{T}y_{k}}{∥g_{k+1}{∥}^{2}},\;\;\beta_{k}^{D}=\max\left\{\beta_{k}^{DHS},\eta_{k}\right\}, \end{array}$$

where

$$\begin{array}{c} \eta_{k}=\frac{-1}{∥d_{k}∥\text{min}\;\{\eta ,∥g_{k}∥\}}, \end{array}$$

and

$$\begin{array}{c} \beta_{k}^{DHS}=(1-\frac{g_{k}^{T}d_{k}}{d_{k}^{T}y_{k}})\beta_{k}^{HS}-t\frac{∥y_{k}{∥}^{2}g_{k+1}^{T}d_{k}}{d_{k}^{T}y_{k}},\;\;t>0. \end{array}$$

Motivated by this approach, Aminifard and Babaie-Kafaki [53] made an almost similar modification to the classical PRP method, which is also a scheme with the same built-in mechanism as the HS method but also fails to satisfy the condition (7) when inexact line searches are employed in general as demonstrated in the case of the HS method. The search direction put forward by the authors in [53] for which (7) holds, while retaining nice attributes of the unmodified PRP method is defined as follows:

$$\begin{array}{c} d_{0}=-g_{0},\;\;d_{k+1}^{M}=\{\begin{array}{c} -\lambda_{k+1}g_{k+1}+\beta_{k}^{MPRP}d_{k},\;g_{k+1}^{T}d_{k}>0,\;k\geq 0;\\ -g_{k+1}+\beta_{k}^{PRP}d_{k},\;g_{k+1}^{T}d_{k}\leq 0,\;k\geq 0, \end{array} \end{array}$$ (19)

where

$$\begin{array}{c} \lambda_{k+1}=1+\frac{g_{k+1}^{T}d_{k}}{∥g_{k+1}{∥}^{2}}\beta_{k}^{PRP}, \end{array}$$

and

$$\begin{array}{c} \beta_{k}^{MPRP}=(1-\frac{g_{k+1}^{T}s_{k}}{∥g_{k}{∥}^{2}})\beta_{k}^{PRP}-t\frac{∥y_{k}{∥}^{2}g_{k+1}^{T}s_{k}}{∥g_{k}{∥}^{4}},\;\;t\geq 0. \end{array}$$

Inspired by (18), (19), and the classical DY method, we propose the following DY-type search directions:

$$\begin{array}{c} d_{0}=-F_{0},\;\;d_{k+1}=\{\begin{array}{c} -\lambda_{k+1}F_{k+1}+\beta_{k}^{MDY1}d_{k},\;F_{k+1}^{T}d_{k}>0,\;k\geq 0;\\ -F_{k+1}+\beta_{k}^{MDY2}d_{k},\;F_{k+1}^{T}d_{k}\leq 0,\;k\geq 0,\;\;\text{otherwise}, \end{array} \end{array}$$ (20)

in which

$$\begin{array}{c} \lambda_{k+1}=1-\gamma_{k+1}\frac{F_{k+1}^{T}d_{k}}{∥F_{k+1}{∥}^{2}}\beta_{k}^{MDY3},\;\;\beta_{k}^{MDY3}=\frac{∥F_{k+1}{∥}^{2}}{d_{k}^{T}w_{k}},\;\;\gamma_{k+1}=\frac{F_{k+1}^{T}d_{k}}{d_{k}^{T}w_{k}}, \end{array}$$ (21)

where

$$\begin{array}{c} \beta_{k}^{MDY1}=\gamma_{k+1}\frac{∥F_{k+1}{∥}^{2}}{d_{k}^{T}w_{k}}-t\frac{∥F_{k+1}{∥}^{2}F_{k+1}^{T}d_{k}}{{\left(d_{k}^{T}w_{k}\right)}^{2}}, \end{array}$$ (22)

with

$$\begin{array}{c} \beta_{k}^{MDY2}=\beta_{k}^{MDY3}-t\frac{∥F_{k+1}{∥}^{2}F_{k+1}^{T}d_{k}}{{\left(d_{k}^{T}w_{k}\right)}^{2}}, \end{array}$$ (23)

and *t* is a positive parameter in the interval (0, 2). Also,

$$\begin{array}{c} w_{k}=y_{k}+\mu \frac{∥F_{k+1}∥s_{k}}{∥s_{k}∥},\;\;s_{k}=z_{k}-x_{k},\;\;y_{k}=F\left(z_{k}\right)-F\left(x_{k}\right),\;\;\mu >0, \end{array}$$ (24)

and

$$\begin{array}{c} z_{k}=x_{k}-\vartheta_{k}d_{k}. \end{array}$$

The parameter $\beta_{k}^{MDY1}$ and $\beta_{k}^{MDY2}$ are the improved Dai-Yuan CG parameters and are both constructed in a similar with the $\beta_{k}^{DHS}$ proposed by Dong et al. [52] and the $\beta_{k}^{MPRP}$ proposed by Aminifard and Babaie-Kafaki [53]. The direction (20) has both the scaling and restarting advantages that made it satisfy the sufficient descent condition without the aid of any line search procedure, and the accelerated convergence rate both numerically and theoretically. From (24) and monotonicity of *F*, we obtain

$$\begin{array}{c} \begin{array}{cc} d_{k}^{T}w_{k} & =d_{k}^{T}y_{k}+\mu \frac{∥F_{k+1}∥}{∥s_{k}∥}d_{k}^{T}s_{k}\\ & =-\frac{s_{k}^{T}y_{k}}{\vartheta_{k}}-\frac{\mu }{\vartheta_{k}}\frac{∥F_{k+1}∥}{∥s_{k}∥}{∥s_{k}∥}^{2}\\ & \geq -\frac{\mu }{\vartheta_{k}}∥F_{k+1}∥∥s_{k}∥\\ & =-\mu ∥F_{k+1}∥∥\frac{s_{k}}{\vartheta_{k}}∥\\ & =-\mu ∥F_{k+1}∥∥d_{k}∥, \end{array} \end{array}$$ (25)

where in the second equality we used the definition of *s*_*k*_ = *z*_*k*_ − *x*_*k*_ = −*ϑ*_*k*_*d*_*k*_, and the inequality follows from the monotonicity of *F*. The steps of the spectral-modified Dai-Yuan algorithm are as follows:

**Algorithm 1 (Spectral Modified Dai-Yuan Method)**

**Initial Data**: Initialized *ϵ* > 0, $x_{0}\in C$, *ζ* ∈ (0, 1), *σ* ∈ (0, 1), *μ* > 2, *t* ∈ (0, 2).

**Initialization**: Set *k* = 0 and *d*_0_ = −*F*_0_.

1: Determine *F*(*x*_*k*_) and verify if ‖*F*(*x*_*k*_)‖ ≤ *ϵ*. If yes, stop; otherwise go to 2.

2: Set *z*_*k*_ = *x*_*k*_ + *ϑ*_*k*_*d*_*k*_, and find $\vartheta_{k}=\zeta^{m_{k}}$, where *m*_*k*_ is the smallest nonnegative integer *m* that satisfies

$$\begin{array}{c} -F{\left(x_{k}+\vartheta_{k}d_{k}\right)}^{T}d_{k}\geq \sigma \vartheta_{k}{∥d_{k}∥}^{2} \end{array}$$ (26)

holds.

3: If $z_{k}\in C$ and ‖*F*(*z*_*k*_)‖ ≤ *ϵ*, stop, otherwise obtain

$$\begin{array}{c} x_{k+1}=P_{C}[x_{k}-\varpi_{k}F\left(z_{k}\right)],\;\;\text{where} \end{array}$$ (27)

$$\begin{array}{c} \varpi_{k}=\frac{F{\left(z_{k}\right)}^{T}\left(x_{k}-z_{k}\right)}{∥F\left(z_{k}\right){∥}^{2}}. \end{array}$$ (28)

4: Determine *d*_*k*+1_ by the formula (20).

5: We repeat the procedure from 1 by setting *k* = *k* + 1 until the stopping condition is satisfied.

## Convergence results

This will give the convergence rate and the global convergence for the spectral-modified Dai-Yuan algorithm. Now, we start with the following helpful ***assumptions***:

1. We assumed $\bar{x}\in C$ exists such that $F(\bar{x})=0$.
2. Given *L* > 1, we have
  $$\begin{array}{c} ∥F(x)-F(y)∥\leq L∥x-y∥,\;\;\forall x,y\in C. \end{array}$$ (29)

Before we proceed, it is required to state an important property of the projection operator which will be recalled later.

**Lemma 1** *Given that* C *is as defined in* (14), *then*

$$\begin{array}{c} ∥P_{C}\left(y\right)-P_{C}\left(x\right)∥\leq ∥y-x∥, \end{array}$$

*and*

$$\begin{array}{c} ∥P_{C}\left(y\right)-x∥\leq ∥y-x∥,\;\forall x,y\in C. \end{array}$$ (30)

**Lemma 2** *The sequence of search directions for the spectral-modified Dai-Yuan algorithm satisfies the inequalities*

$$\begin{array}{c} d_{k+1}^{T}F_{k+1}\leq -c{∥F_{k+1}∥}^{2},\;\;c=\text{min}\;\left\{1,1-\frac{2-t}{\mu^{2}}\right\}, \end{array}$$ (31)

*where μ* > 2, *and t* ∈ (0, 2).

**Proof**: It can be seen from (20) that for *k* = 0, $d_{0}^{T}F_{0}=-F_{0}^{T}\left(F_{0}\right)=-{∥F_{0}∥}^{2}$. Next, we analyze the two cases presented in (20) for *k* = 1, 2, ….

First case: $F_{k+1}^{T}d_{k}>0$. By pre-multiplying (20) by *F*_*k*+1_, we get

$$\begin{array}{c} \begin{array}{cc} d_{k+1}^{T}F_{k+1} & =-(1-\gamma_{k+1}\frac{F_{k+1}^{T}d_{k}}{∥F_{k+1}{∥}^{2}}\beta_{k}^{MDY3}){∥F_{k+1}∥}^{2}+\gamma_{k+1}\frac{∥F_{k+1}{∥}^{2}F_{k+1}^{T}d_{k}}{d_{k}^{T}w_{k}}-t\frac{∥F_{k+1}{∥}^{2}{\left(F_{k+1}^{T}d_{k}\right)}^{2}}{{\left(d_{k}^{T}w_{k}\right)}^{2}}\\ & =-∥F_{k+1}{∥}^{2}+\gamma_{k+1}\frac{∥F_{k+1}{∥}^{2}F_{k+1}^{T}d_{k}}{d_{k}^{T}w_{k}}+\gamma_{k+1}\frac{∥F_{k+1}{∥}^{2}F_{k+1}^{T}d_{k}}{d_{k}^{T}w_{k}}-t\frac{∥F_{k+1}{∥}^{2}{\left(F_{k+1}^{T}d_{k}\right)}^{2}}{{\left(d_{k}^{T}w_{k}\right)}^{2}}\\ & =-∥F_{k+1}{∥}^{2}+2\frac{∥F_{k+1}{∥}^{2}{\left(F_{k+1}^{T}d_{k}\right)}^{2}}{{\left(d_{k}^{T}w_{k}\right)}^{2}}-t\frac{∥F_{k+1}{∥}^{2}{\left(F_{k+1}^{T}d_{k}\right)}^{2}}{{\left(d_{k}^{T}w_{k}\right)}^{2}}\\ & =-∥F_{k+1}{∥}^{2}+\frac{{\left(F_{k+1}^{T}d_{k}\right)}^{2}}{{\left(d_{k}^{T}w_{k}\right)}^{2}}\left(2-t\right){∥F_{k+1}∥}^{2}\\ & \leq -∥F_{k+1}{∥}^{2}+\frac{∥F_{k+1}{∥}^{2}{∥d_{k}∥}^{2}}{\mu^{2}∥F_{k+1}{∥}^{2}{∥d_{k}∥}^{2}}\left(2-t\right){∥F_{k+1}∥}^{2}\\ & \leq -∥F_{k+1}{∥}^{2}+\frac{\left(2-t\right)}{\mu^{2}}{∥F_{k+1}∥}^{2}\\ & \leq -(1-\frac{\left(2-t\right)}{\mu^{2}}){∥F_{k+1}∥}^{2},\;\;\mu >2,\;\;t\in \left(0,2\right) \end{array} \end{array}$$ (32)

Second case: $F_{k+1}^{T}d_{k}\leq 0$. Given that ‖*F*_*k*+1_‖^2^ and $d_{k}^{T}w_{k}$ are both greater than zero, pre-multiplying the *d*_*k*+1_ for the second case by *F*_*k*+1_, we obtain

$$\begin{array}{c} d_{k+1}^{T}F_{k+1}=-∥F_{k+1}{∥}^{2}+\frac{∥F_{k+1}{∥}^{2}F_{k+1}^{T}d_{k}}{d_{k}^{T}w_{k}}-t\frac{∥F_{k+1}{∥}^{2}{\left(F_{k+1}^{T}d_{k}\right)}^{2}}{{\left(d_{k}^{T}w_{k}\right)}^{2}}\leq -{∥F_{k+1}∥}^{2}. \end{array}$$ (33)

Therefore, utilizing (32) and (33), we obtain the inequality in (31), which establishes the proof.

Now, from Cauchy Schwartz inequality (16) and (31), we obtain the first inequality. It can also be seen from that from (20) and for *k* = 0, *d*_0_ = −*F*_0_ which implies that ‖*d*_0_‖ = ‖*F*_0_‖. Now, for *k* = 1, 2, …, we analyze two cases.

First case: $F_{k+1}^{T}d_{k}>0$. From (20), (21), (22), (25), and (16), we have

$$\begin{array}{c} \begin{array}{cc} ∥d_{k+1}∥ & =∥-\lambda_{k+1}F_{k+1}+\gamma_{k+1}\frac{∥F_{k+1}{∥}^{2}}{d_{k}^{T}w_{k}}d_{k}-t\frac{∥F_{k+1}{∥}^{2}F_{k+1}^{T}d_{k}}{{\left(d_{k}^{T}w_{k}\right)}^{2}}d_{k}∥\\ & =∥-F_{k+1}+\gamma_{k+1}\frac{F_{k+1}^{T}d_{k}}{∥F_{k+1}{∥}^{2}}\frac{∥F_{k+1}{∥}^{2}}{d_{k}^{T}w_{k}}F_{k+1}+\gamma_{k+1}\frac{∥F_{k+1}{∥}^{2}}{d_{k}^{T}w_{k}}d_{k}-t\frac{∥F_{k+1}{∥}^{2}F_{k+1}^{T}d_{k}}{{\left(d_{k}^{T}w_{k}\right)}^{2}}d_{k}∥\\ & =∥-F_{k+1}+\frac{{\left(F_{k+1}^{T}d_{k}\right)}^{2}}{{\left(d_{k}^{T}w_{k}\right)}^{2}}F_{k+1}+\frac{F_{k+1}^{T}d_{k}{∥F_{k+1}∥}^{2}}{{\left(d_{k}^{T}w_{k}\right)}^{2}}d_{k}-t\frac{∥F_{k+1}{∥}^{2}F_{k+1}^{T}d_{k}}{{\left(d_{k}^{T}w_{k}\right)}^{2}}d_{k}∥\\ & \leq ∥F_{k+1}∥+\frac{∥F_{k+1}{∥}^{3}{∥d_{k}∥}^{2}}{{\left(d_{k}^{T}w_{k}\right)}^{2}}+\frac{∥F_{k+1}{∥}^{3}{∥d_{k}∥}^{2}}{{\left(d_{k}^{T}w_{k}\right)}^{2}}+t\frac{∥F_{k+1}{∥}^{3}{∥d_{k}∥}^{2}}{{\left(d_{k}^{T}w_{k}\right)}^{2}}\\ & \leq ∥F_{k+1}∥+\frac{∥F_{k+1}{∥}^{3}{∥d_{k}∥}^{2}}{\mu^{2}∥F_{k+1}{∥}^{2}{∥d_{k}∥}^{2}}+\frac{∥F_{k+1}{∥}^{3}{∥d_{k}∥}^{2}}{\mu^{2}∥F_{k+1}{∥}^{2}{∥d_{k}∥}^{2}}+t\frac{∥F_{k+1}{∥}^{3}{∥d_{k}∥}^{2}}{\mu^{2}∥F_{k+1}{∥}^{2}{∥d_{k}∥}^{2}}\\ & =∥F_{k+1}∥+\frac{∥F_{k+1}∥}{\mu^{2}}+\frac{∥F_{k+1}∥}{\mu^{2}}+t\frac{∥F_{k+1}∥}{\mu^{2}}\\ & =(1+\frac{2}{\mu^{2}}+\frac{t}{\mu^{2}})∥F_{k+1}∥. \end{array} \end{array}$$ (34)

Case (2) $F_{k+1}^{T}d_{k}\leq 0$. From (20), (23) and (25), we have

$$\begin{array}{c} \begin{array}{cc} ∥d_{k+1}∥ & =∥-F_{k+1}+\frac{∥F_{k+1}{∥}^{2}}{d_{k}^{T}w_{k}}d_{k}-t\frac{∥F_{k+1}{∥}^{2}F_{k+1}^{T}d_{k}}{{\left(d_{k}^{T}w_{k}\right)}^{2}}d_{k}∥\\ & \leq ∥F_{k+1}∥+\frac{∥F_{k+1}{∥}^{2}∥d_{k}∥}{d_{k}^{T}w_{k}}+t\frac{∥F_{k+1}{∥}^{3}{∥d_{k}∥}^{2}}{{\left(d_{k}^{T}w_{k}\right)}^{2}}\\ & \leq ∥F_{k+1}∥-\frac{∥F_{k+1}{∥}^{2}∥d_{k}∥}{\mu ∥F_{k+1}∥∥d_{k}∥}+t\frac{∥F_{k+1}{∥}^{3}{∥d_{k}∥}^{2}}{\mu^{2}∥F_{k+1}{∥}^{2}{∥d_{k}∥}^{2}}\\ & =∥F_{k+1}∥+\frac{∥F_{k+1}∥}{\mu }+t\frac{∥F_{k+1}∥}{\mu^{2}}\\ & =(1+\frac{1}{\mu }+\frac{t}{\mu^{2}})∥F_{k+1}∥\\ . \end{array} \end{array}$$ (35)

Hence, from (34) and (35), we get

$$\begin{array}{c} ∥d_{k+1}∥\leq (1+\frac{1}{\mu }+\frac{t}{\mu^{2}})∥F_{k+1}∥. \end{array}$$ (36)

This Lemma will be used to illustrate the next Lemma result.

**Lemma 3** *(1) Let d*_*k*_ *and x*_*k*_ *be sequences generated using the spectral-modified Dai-Yuan algorithm. Then a non-negative integer exists such that* (26) *holds*.

*(2) Assuming that Assumption 2 holds true for* {*x*_*k*_} *and* {*z*_*k*_} *obtained using the spectral-modified Dai-Yuan algorithm. Then α*_*k*_ > 0 *satisfies*

$$\begin{array}{c} \vartheta_{k}\geq \vartheta :=\text{min}\;\{1,\frac{\zeta c}{\left(L+\sigma \right){\left[1+\frac{1}{\mu }+\frac{t}{\mu }\right]}^{2}}\}. \end{array}$$ (37)

**Proof**: Proof of (1) is omitted since it has been proven in [54]. To show (2), we assume that Algorithm 1 terminates at some point *x*_*k*_. If so, it follows that *F*(*z*_*k*_) = 0 or *F*(*x*_*k*_) = 0. If *F*(*x*_*k*_) ≠ 0, then the direction *d*_*k*_ ≠ 0 as well. We will demonstrate that (26) always stops within finite iterations. If *F*(*x*_*k*_) ≠ 0, then *d*_*k*_ ≠ 0 follows from (31). We now demonstrate that the line search (26) always ends within finite iterations. Suppose *ϑ*_*k*_ = 1 in (26), then (26) holds, otherwise, ${\bar{\vartheta }}_{k}=\zeta^{-1}\vartheta_{k}$ will not satisfy (26), namely,

$$\begin{array}{c} -F{\left({\bar{z}}_{k}\right)}^{T}d_{k}<\sigma {\bar{\vartheta }}_{k}{∥d_{k}∥}^{2}, \end{array}$$

with, ${\bar{z}}_{k}=x_{k}+{\bar{\vartheta }}_{k}d_{k}$. From Assumption 2 and (26), we can write

$$\begin{array}{c} \begin{array}{cc} c∥F_{k}{∥}^{2} & \leq -F_{k}^{T}d_{k}={\left(F\left({\bar{z}}_{k}\right)-F_{k}\right)}^{T}d_{k}-F{\left({\bar{z}}_{k}\right)}^{T}d_{k}\\ & <{\bar{\vartheta }}_{k}\left(L+\sigma \right){∥d_{k}∥}^{2}\\ & =\zeta^{-1}\vartheta_{k}\left(L+\sigma \right){∥d_{k}∥}^{2}. \end{array} \end{array}$$

Therefore, we have

$$\begin{array}{c} \begin{array}{cc} \vartheta_{k} & >\frac{\zeta c∥F_{k}{∥}^{2}}{\left(L+\sigma \right)∥d_{k}{∥}^{2}}\\ & \geq \frac{\zeta c∥F_{k}{∥}^{2}}{\left(L+\sigma \right)}\frac{∥F_{k}{∥}^{2}}{{\left(1+\frac{1}{\mu }+\frac{t}{\mu^{2}}\right)}^{2}{∥F_{k}∥}^{4}}\\ & =\frac{\zeta c}{\left(L+\sigma \right){\left[1+\frac{1}{\mu }+\frac{t}{\mu }\right]}^{2}}, \end{array} \end{array}$$

with the second inequality obtained from (36).

**Lemma 4** *Assume that Assumptions* 1 *and* 2 *are true. Therefore* $\left\{x_{k}-\bar{x}\right\}$ *is convergent for any arbitrary solution* $\bar{x}$ in $\bar{C}$ *and consequently*

$$\begin{array}{c} \underset{k\to \infty }{\text{lim}}\;\vartheta_{k}∥d_{k}∥=0. \end{array}$$ (38)

**Proof**: To begin the proof, we start with the boundedness of {*x*_*k*_} and {*z*_*k*_}. From (15) and *z*_*k*_, we obtain

$$\begin{array}{c} {\left(x_{k}-z_{k}\right)}^{T}F\left(z_{k}\right)\geq \sigma \vartheta_{k}^{2}{∥d_{k}∥}^{2}. \end{array}$$ (39)

From (15) and $\bar{x}\in \bar{C}$, we get

$$\begin{array}{c} \begin{array}{cc} {\left(x_{k}-\bar{x}\right)}^{T}F\left(z_{k}\right) & ={\left(x_{k}-z_{k}\right)}^{T}F\left(z_{k}\right)+{\left(z_{k}-\bar{x}\right)}^{T}F\left(z_{k}\right)\\ & \geq {\left(x_{k}-z_{k}\right)}^{T}F\left(z_{k}\right)+{\left(z_{k}-\bar{x}\right)}^{T}F\left(\bar{x}\right)\\ & ={\left(x_{k}-z_{k}\right)}^{T}F\left(z_{k}\right). \end{array} \end{array}$$ (40)

Utilizing (30), (27), (28), (39) and (40), we have

$$\begin{array}{c} \begin{array}{cc} ∥x_{k+1}-\bar{x}{∥}^{2} & =∥P_{C}\left[x_{k}-\varpi_{k}F\left(z_{k}\right)\right]-\bar{x}{∥}^{2}\\ & \leq ∥x_{k}-\varpi_{k}F\left(z_{k}\right)-\bar{x}{∥}^{2}\\ & =∥\left(x_{k}-\bar{x}\right)-\varpi_{k}F\left(z_{k}\right){∥}^{2}\\ & =∥x_{k}-\bar{x}{∥}^{2}-2\varpi_{k}F{\left(z_{k}\right)}^{T}\left(x_{k}-\bar{x}\right)+\varpi_{k}^{2}{∥F\left(z_{k}\right)∥}^{2}\\ & \leq ∥x_{k}-\bar{x}{∥}^{2}-2\varpi_{k}F{\left(z_{k}\right)}^{T}\left(x_{k}-z_{k}\right)+\varpi_{k}^{2}{∥F\left(z_{k}\right)∥}^{2}\\ & =∥x_{k}-\bar{x}{∥}^{2}-\frac{{\left(F{\left(z_{k}\right)}^{T}\left(x_{k}-z_{k}\right)\right)}^{2}}{∥F\left(z_{k}\right){∥}^{2}}\\ & \leq ∥x_{k}-\bar{x}{∥}^{2}-\frac{\sigma^{2}{∥x_{k}-z_{k}∥}^{4}}{∥F\left(z_{k}\right){∥}^{2}}. \end{array} \end{array}$$ (41)

So that we obtain

$$\begin{array}{c} 0\leq ∥x_{k+1}-\bar{x}∥\leq ∥x_{k}-\bar{x}∥,\;\forall k\geq 0. \end{array}$$

Therefore, $\left\{∥x_{k}-\bar{x}∥\right\}$ is a decreasing sequence which shows it is bounded and {*x*_*k*_} is also bounded. From the boundedness of {*x*_*k*_} and continuity of *F*, we have that there exists a constant *ξ*_1_ such that for all *k* ≥ 0,

$$\begin{array}{c} ∥x_{k}∥\leq \xi_{1},\;\;∥F\left(x_{k}\right)∥\leq \xi_{1}. \end{array}$$ (42)

Also, from (36) and (42), we obtain

$$\begin{array}{c} ∥d_{k+1}∥\leq (1+\frac{1}{\mu }+\frac{t}{\mu^{2}})∥F_{k+1}∥\leq (1+\frac{1}{\mu }+\frac{t}{\mu^{2}})\xi_{1}. \end{array}$$

Hence, there exists a constant *ξ*_2_ such that

$$\begin{array}{c} ∥d_{k+1}∥\leq \xi_{2}, \end{array}$$

where $\xi_{2}=(1+\frac{1}{\mu }+\frac{t}{\mu^{2}})∥F_{k+1}∥\leq (1+\frac{1}{\mu }+\frac{t}{\mu^{2}})\xi_{1}$. From (15), (16), (39) and (42), we have

$$\begin{array}{c} \xi_{1}\geq ∥F_{k}∥\geq \frac{F_{k}^{T}\left(x_{k}-z_{k}\right)}{∥x_{k}-z_{k}∥}\geq \frac{F{\left(z_{k}\right)}^{T}\left(x_{k}-z_{k}\right)}{∥x_{k}-z_{k}∥}\geq \sigma ∥x_{k}-z_{k}∥\geq \sigma ∥z_{k}∥-\sigma \xi_{1}, \end{array}$$

which further implies that

$$\begin{array}{c} ∥z_{k}∥\leq \frac{\xi_{1}+\sigma \xi_{1}}{\sigma }. \end{array}$$

By setting $\xi_{3}:=\frac{\xi_{1}+\sigma \xi_{1}}{\sigma }$, the boundedness of {*z*_*k*_} is established. Also, the continuity of *F*, implies that there exists a constant $\bar{\psi }$ such that

$$\begin{array}{c} ∥F\left(z_{k}\right)∥\leq \bar{\psi }.\;\;\forall k\geq 0. \end{array}$$

This with (41) yields

$$\begin{array}{c} \sigma^{2}∥x_{k}-z_{k}{∥}^{4}\leq {\bar{\psi }}^{2}(∥x_{k}-\bar{x}{∥}^{2}-∥x_{k+1}-\bar{x}{∥}^{2}). \end{array}$$ (43)

Considering that $\left\{∥x_{k}-\bar{x}∥\right\}$ is convergent and {*F*(*z*_*k*_)} is bounded, we have from (43) that

$$\begin{array}{c} \sigma^{2}\underset{k\;\to \;\infty }{\text{lim}}\vartheta_{k}^{4}{∥d_{k}∥}^{4}\leq 0, \end{array}$$

which leads to

$$\begin{array}{c} \underset{k\;\to \;\infty }{\text{lim}}\vartheta_{k}∥d_{k}∥=0. \end{array}$$

**Theorem 5** *Considering the Assumptions* 1 *and* 2, *the sequence* {*x*_*k*_} *converges*.

**Proof**: Utilizing (37) and (38), we see that when 0 ≤ *ϑ*‖*d*_*k*_‖ ≤ *ϑ*_*k*_‖*d*_*k*_‖ → 0. In addition, lim_*k*→∞_‖*d*_*k*_‖ = 0. From (31), it yields the relation

$$\begin{array}{c} 0\leq c∥F_{k}∥\leq ∥d_{k}∥\to 0,\;\;c=\text{min}\;\left\{1,1-\frac{2-t}{\mu^{2}}\right\},\;\;\mu >2,\;\;t\in \left(0,2\right). \end{array}$$

This indicates that lim_*k*→∞_‖*F*_*k*_‖ = 0. From (38) and boundedness of {*x*_*k*_}, we have that a cluster point of {*x*_*k*_} exists. Suppose $\tilde{x}$ represents a cluster point of $\left\{x_{k}\right\}\subset \bar{C}$, where K⊂{0,1,2,…} is an indexing set for which

$$\begin{array}{c} \underset{k\;\to \;\infty ,k\in K}{\text{lim}}x_{k}=\tilde{x_{k}}\in \bar{C}. \end{array}$$

Then, by continuity of *F*, we get

$$\begin{array}{c} 0=\underset{k\;\to \;\infty }{\text{lim}}∥F_{k}∥=\underset{k\;\to \;\infty ,k\in K}{\text{lim}}∥F_{k}∥=∥F\left(\tilde{x}\right)∥, \end{array}$$

which implies that $\tilde{x}$ is a solution of (14), i.e., $\tilde{x}\in \bar{C}$. Furthermore, $\left\{x_{k}-\bar{x}\right\}$ is convergent from Lemma 3.3. Therefore, setting $\bar{x}=\tilde{x}$, we obtain

$$\begin{array}{c} \underset{k\;\to \;\infty }{\text{lim}}∥x_{k}-\bar{x}∥=\underset{k\;\to \;\infty ,k\in K}{\text{lim}}∥x_{k}-\bar{x}∥=0. \end{array}$$

So, we conclude that the sequence {*x*_*k*_} goes to $\bar{x}\in \bar{C}$.

**Assumption 3**. Given that *τ* ∈ (0, 1), ς>0, and $\bar{x}\in \bar{C}$ satisfying

$$\begin{array}{c} \tau \text{dist}\left(x,\bar{C}\right)\leq ∥F\left(x\right)∥,\;\;\forall x\in N_{\varsigma }\left(\bar{x}\right), \end{array}$$ (44)

where $N_{\varsigma }\left(\bar{x}\right)$ stands for the neighbourhood of $\bar{x}$, given as

$$\begin{array}{c} N_{\varsigma }\left(\bar{x}\right):=\{x\in R^{n}:∥x-\bar{x}∥\leq \varsigma \}. \end{array}$$

Thus, $\text{dist}(x,\bar{C})$ specifies the distance between *x* and $\bar{C}$.

**Theorem 6** *If Assumptions* 1, 2, *and* 3 *are true for* {*x*_*k*_} *given by Algorithm* 1, *then* $dist(x_{k},\bar{C})$ *converges Q* − *linearly to* 0, *implying that* {*x*_*k*_} *converges to* $\bar{x}$ *R* − *linearly*.

**Proof**: Suppose that ${\tilde{x}}_{k}:=argmin\{∥x_{k}-\tilde{x}∥:\tilde{x}\in \bar{C}\}$. Then

$$\begin{array}{c} ∥x_{k}-{\tilde{x}}_{k}∥=\text{dist}\left(x_{k},\bar{C}\right). \end{array}$$ (45)

By replacing $\bar{x}$ with $\tilde{x}$ in (41), we have

$$\begin{array}{c} ∥x_{k+1}-\tilde{x}{∥}^{2}\leq {∥x_{k}-\tilde{x}∥}^{2}-\frac{\sigma^{2}\vartheta_{k}^{4}{∥d_{k}∥}^{4}}{∥F\left(z_{k}\right){∥}^{2}}. \end{array}$$ (46)

From (29), (36), (45) and since for a solution ${\tilde{x}}_{k}$ $\in \bar{C}$, $F({\tilde{x}}_{k})=0$, we can write

$$\begin{array}{c} \begin{array}{cc} ∥F(z_{k})∥ & =∥F\left(z_{k}\right)-F\left({\tilde{x}}_{k}\right)∥\leq L∥z_{k}-{\tilde{x}}_{k}∥\leq L(∥z_{k}-x_{k}∥+∥x_{k}-{\tilde{x}}_{k}∥)\\ & =L(\vartheta_{k}∥d_{k}∥+∥x_{k}-{\tilde{x}}_{k}∥)\leq L(∥d_{k}∥+∥x_{k}-{\tilde{x}}_{k}∥)\\ & \leq L((1+\frac{1}{\mu }+\frac{t}{\mu^{2}})∥F\left(x_{k}\right)∥+∥x_{k}-{\tilde{x}}_{k}∥)\\ & =L((1+\frac{1}{\mu }+\frac{t}{\mu^{2}})∥F\left(x_{k}\right)-F\left({\tilde{x}}_{k}\right)∥+∥x_{k}-{\tilde{x}}_{k}∥)\\ & \leq L(1+L(1+\frac{1}{\mu }+\frac{t}{\mu^{2}}))∥x_{k}-{\tilde{x}}_{k}∥\\ & =L(1+L(1+\frac{1}{\mu }+\frac{t}{\mu^{2}}))\text{dist}\left(x_{k},\bar{C}\right)\\ & =L\left(1+L\varphi \right)\text{dist}\left(x_{k},\bar{C}\right), \end{array} \end{array}$$ (47)

where $\varphi =(1+\frac{1}{\mu }+\frac{t}{\mu^{2}})$.

Next, from (31) and Cauchy Schwartz inequality (16) by assuming *c* = 1 to have

$$\begin{array}{c} ∥d_{k}∥\geq ∥F_{k}∥. \end{array}$$ (48)

Thus, since ${\tilde{x}}_{k}\in \bar{C}$, using (44), (45), (46), (47), (48), and (37), we obtain

$$\begin{array}{c} \begin{array}{cc} \text{dist}{\left(x_{k+1},\bar{C}\right)}^{2} & =∥x_{k+1}-{\tilde{x}}_{k}{∥}^{2}\\ & \leq \text{dist}{\left(x_{k},\bar{C}\right)}^{2}-\frac{\sigma^{2}\vartheta_{k}^{4}{∥d_{k}∥}^{4}}{∥F\left(z_{k}\right){∥}^{2}}\\ & \leq \text{dist}{\left(x_{k},\bar{C}\right)}^{2}-\frac{\sigma^{2}\vartheta^{4}{∥F\left(x_{k}\right)∥}^{4}}{∥F(z_{k}{∥}^{2}}\\ & \leq \text{dist}{\left(x_{k},\bar{C}\right)}^{2}-\frac{\sigma^{2}\vartheta^{4}\tau^{4}\text{dist}{\left(x_{k},\bar{C}\right)}^{4}}{L^{2}{\left(L\varphi +1\right)}^{2}\text{dist}{\left(x_{k},\bar{C}\right)}^{2}}\\ & =(1-\frac{\sigma^{2}\vartheta^{4}\tau^{4}}{L^{2}{\left(L\varphi +1\right)}^{2}})\text{dist}{\left(x_{k},\bar{C}\right)}^{2}. \end{array} \end{array}$$

Since *τ*, *σ*, *ϑ* ∈ (0, 1), and *L* > 1, *φ* > 1; then $\frac{\sigma^{2}\vartheta^{4}\tau^{4}}{L^{2}{\left(L\varphi +1\right)}^{2}}<1$. It follows that $\left\{\text{dist}\left(x_{k},\bar{C}\right)\right\}$ converges Q-linearly to 0, meaning that {*x*_*k*_} is R-linearly convergent to $\bar{x}$.

## Numerical results

In this part, we give the numerical findings for the spectral-modified Dai-Yuan algorithm to demonstrate its usefulness, which we identify as SDYM. The proposed algorithm is evaluated via some current spectral-like methods from the literature:

- A method for solving nonlinear monotone equations using scaled gradient projections (SCGP) [55].
- The prominent spectral CG DY-type for NCMS (MDY) [56].
- A convex constraints RMIL CG method for monotone equations (SRMIL) [57].

We choose to compare the SDYM algorithm with these algorithms because they are also derivative-free algorithms with good convergence properties and are numerically efficient, for more details on the compared algorithms, we refer readers to the highlighted references. The four algorithms are written in Matlab R2015 on a PC with the following specifications (4 GB RAM, 2.30 GHZ CPU). The methods are also implemented using the line search given in (26), with the SCGP, MDY, and SRMIL parameters applied as they appear in each of the papers. Parameters for SDYM are given as *ζ* = 0.4, *σ* = 10^−3^, *ψ* = 1.9, *μ* = 2.5, *t* = 0.6. Each Matlab program completes when ‖*F*(*x*_*k*_)‖ ≤ 10^−10^ or ‖*F*(*z*_*k*_)‖ ≤ 10^−10^ or 1000 iterations are reached. Six examples of the operator *F*, which are presented as Example 1 to Example 6 are chosen for the experiments with dimensions 10^3^, 10^4^, 5 × 10^4^ and the following initial starting points:

$x_{0}^{1}={\left(\frac{1}{n},\frac{2}{n},...,1\right)}^{T}$ , $x_{0}^{2}={\left(\frac{3}{2},\frac{1}{2},\ldots ,-\frac{\left[{\left(-1\right)}^{n}-2\right]}{2}\right)}^{T}$, $x_{0}^{3}={\left(3,2,\ldots ,\ldots ,-\frac{\left[{\left(-1\right)}^{n}-5\right]}{2}\right)}^{T}$, $x_{0}^{4}={\left(\frac{5}{2},\frac{3}{2},\ldots ,-\frac{\left[{\left(-1\right)}^{n}-4\right]}{2}\right)}^{T}$, $x_{0}^{5}={\left(3,1,\ldots ,-2\frac{\left[{\left(-1\right)}^{n}-2\right]}{2}\right)}^{T}$, $x_{0}^{6}={\left(2,1,\ldots ,-\frac{\left[{\left(-1\right)}^{n}-3\right]}{2}\right)}^{T}$

The numerical findings using six examples of monotone operator equations are given by considering C as a convex-constrained set. The examples are as follows:

**Example 1.** This is a Nonsmooth Function that can be found in [58].

*F*_*i*_(*x*) = 2*x*_*i*_ − sin|*x*_*i*_|, *i* = 1, 2, …, *n*,

where $C=R_{+}^{n}$.

**Example 2.** This is a Nonsmooth Function that can be found in [59].

$F_{i}\left(x\right)=\text{log}\left(1+x_{i}\right)-\frac{x_{i}}{n}$ , *i* = 2, …*n*

where $C=R_{+}^{n}$.

**Example 3.** This is a Penalty Function 1 that can be found in [50].

$F_{i}\left(x\right)=2\pi \left(x_{i}-1\right)+x_{i}\sum_{j=1}^{n}4\left(x_{j}-0.25\right),\;i=1,2,\ldots ,n$ ,

where $\pi =10^{-5},\;C=R_{+}^{n}$.

**Example 4.** This is a Nonsmooth Function that can be found in [47].

*F*_*i*_(*x*) = 2*x*_*i*_ − sin|1 − *x*_*i*_|, *i* = 1, 2, …, *n*,

where $C=R_{+}^{n}$.

**Example 5.** This is an artificial problem that can be found in [60].

*F*_1_(*x*) = *x*_2_ − 1 + 2.5*x*_1_,

*F*_*i*_(*x*) = 2.5*x*_*i*_ + *x*_*i*+1_ + *x*_*i*−1_ − 1,

*F*_*n*_(*x*) = 2.5*x*_*n*_ + *x*_*n*−1_ − 1 *i* = 2, 3, …, *n* − 1,

where $C=R_{+}^{n}$.

**Example 6.** This is an artificial problem that can be found in [61].

*F*_1_(*x*) = sin *x*_1_ − 1 + 2*x*_1_,

*F*_*i*_(*x*) = 2 sin*x*_*i*_ + 2*x*_*i*_ + 2*x*_*i*−1_ − 1,

*F*_*n*_(*x*) = 2*x*_*n*_ + sin *x*_*n*_ − 1, *i* = 2, …, *n* − 1,

where $C=R_{+}^{n}$.

The numerical findings carried out for the considered algorithms are given by Tables 1–3, this reads as follows as to what each column’s labels mean: “VAR” and “PN” stand for the dimension and number of the example solved, “IG” and “NOI” are the initial guess and number of iterations respectively. “FV” and “Ptime” denote function values and the processing time recorded in seconds, while “Norm” and “***” stand for the termination point and failure of an algorithm after reaching the maximum iteration (1000).

**Table 1:** SDYM against SCGP, SRMIL, and MDY numerical results for examples 1 and 2.

PN	VAR	IG	SDYM				SCGP				MDY				SRMIL			
			NOI	FV	Ptime	Norm	NOI	FV	Ptime	Norm	NOI	FV	Ptime	Norm	NOI	FV	Ptime	Norm
1	10^3^	$x_{0}^{1}$	12	27	0.0989	5.60E-11	32	123	0.0522	9.73E-11	18	44	0.1598	2.45E-11	22	70	0.1385	6.58E-11
	10^3^	$x_{0}^{2}$	12	25	0.0152	3.55E-11	18	37	0.0197	9.05E-11	16	35	0.0210	2.29E-11	24	65	0.0339	8.94E-11
	10^3^	$x_{0}^{3}$	1	3	0.0037	0	2	5	0.0049	0	16	37	0.0230	3.09E-11	27	81	0.0313	3.89E-11
	10^3^	$x_{0}^{4}$	1	3	0.0038	0	19	39	0.0148	1.00E-10	16	35	0.0288	3.21E-11	25	67	0.0340	4.31E-11
	10^3^	$x_{0}^{5}$	1	3	0.0036	0	2	5	0.0093	0	16	40	0.0238	9.85E-11	29	84	0.0495	4.77E-11
	10^3^	$x_{0}^{6}$	1	3	0.0045	0	21	63	0.0154	5.68E-11	19	51	0.0484	2.89E-11	27	73	0.0410	7.29E-11
	10^4^	$x_{0}^{1}$	13	27	0.0632	3.90E-11	28	89	0.1383	7.13E-11	18	47	0.1423	8.56E-11	23	73	0.1927	6.50E-11
	10^4^	$x_{0}^{2}$	12	27	0.0594	5.93E-11	29	91	0.1345	9.55E-11	17	39	0.1344	2.26E-11	25	68	0.1962	9.61E-11
	10^4^	$x_{0}^{3}$	1	3	0.0083	0	2	5	0.0122	0	17	40	0.1186	4.08E-11	28	84	0.2052	3.94E-11
	10^4^	$x_{0}^{4}$	1	3	0.0105	0	23	92	0.1398	1.66E-11	16	35	0.1151	5.63E-11	26	70	0.1864	3.02E-11
	10^4^	$x_{0}^{5}$	1	3	0.0087	0	2	5	0.0141	0	17	42	0.1244	4.78E-11	30	87	0.2488	4.82E-11
	10^4^	$x_{0}^{6}$	1	3	0.0097	0	33	126	0.1802	6.90E-11	19	50	0.1519	5.91E-11	29	79	0.1966	3.64E-11
	5 × 10^4^	$x_{0}^{1}$	13	27	0.2385	8.72E-11	20	60	0.3709	0	19	47	0.5638	3.25E-11	24	76	0.6661	4.64E-11
	5 × 10^4^	$x_{0}^{2}$	13	27	0.2353	2.63E-11	31	123	0.6523	1.02E-11	17	39	0.4251	3.90E-11	26	71	0.6679	6.92E-11
	5 × 10^4^	$x_{0}^{3}$	1	3	0.0299	0	2	5	0.0454	0	17	40	0.4408	3.80E-11	28	84	0.6967	8.80E-11
	5 × 10^4^	$x_{0}^{4}$	1	3	0.0348	0	32	96	0.5790	8.29E-11	16	35	0.4027	3.97E-11	26	70	0.6552	6.74E-11
	5 × 10^4^	$x_{0}^{5}$	1	3	0.0286	0	2	5	0.0570	0	18	45	0.4886	2.23E-11	31	90	0.8106	3.44E-11
	5 × 10^4^	$x_{0}^{6}$	1	3	0.0308	0	31	115	0.6306	7.83E-11	20	56	0.5513	4.36E-11	29	79	0.7170	8.15E-11
2	10^3^	$x_{0}^{1}$	12	25	0.0161	7.95E-11	47	131	0.0362	5.81E-12	18	44	0.0417	5.01E-11	28	69	0.0551	2.11E-11
	10^3^	$x_{0}^{2}$	12	26	0.0111	5.95E-11	16	33	0.0129	5.69E-11	18	52	0.0366	3.20E-11	24	70	0.0422	6.54E-11
	10^3^	$x_{0}^{3}$	13	26	0.0114	3.21E-11	18	36	0.0199	6.07E-11	20	50	0.0366	6.92E-11	24	70	0.0438	7.73E-11
	10^3^	$x_{0}^{4}$	12	24	0.0113	4.00E-11	2	3	0.0049	0	18	43	0.0288	4.54E-11	24	66	0.0301	5.97E-11
	10^3^	$x_{0}^{5}$	13	26	0.0235	4.89E-11	4	5	0.0064	0	***	***	***	***	24	72	0.0281	3.20E-11
	10^3^	$x_{0}^{6}$	12	24	0.0111	4.75E-11	2	3	0.0054	0	18	47	0.0386	6.70E-11	25	71	0.0593	3.02E-11
	10^4^	$x_{0}^{1}$	12	26	0.0737	6.30E-11	37	108	0.1983	3.87E-11	18	42	0.1611	3.77E-11	23	58	0.1813	9.16E-11
	10^4^	$x_{0}^{2}$	13	26	0.0751	3.40E-11	16	31	0.1002	4.70E-11	19	50	0.1835	3.05E-11	25	70	0.1966	4.64E-11
	10^4^	$x_{0}^{3}$	13	26	0.0728	9.44E-11	31	100	0.1739	7.30E-11	19	44	0.1712	4.86E-11	26	73	0.2229	9.52E-11
	10^4^	$x_{0}^{4}$	12	26	0.0726	6.09E-11	2	3	0.0118	0	19	46	0.1660	3.66E-11	26	71	0.2056	4.61E-11
	10^4^	$x_{0}^{5}$	13	28	0.0800	7.91E-11	4	5	0.0232	0	97	879	3.5741	7.15E-11	25	75	0.2389	3.29E-11
	10^4^	$x_{0}^{6}$	12	26	0.0720	7.43E-11	2	3	0.0136	0	19	47	0.1746	5.00E-11	30	70	0.2403	3.10E-11
	5 × 10^4^	$x_{0}^{1}$	13	26	0.2677	2.64E-11	37	107	0.7887	9.96E-11	19	44	0.5583	4.19E-11	27	73	0.8192	7.74E-11
	5 × 10^4^	$x_{0}^{2}$	13	26	0.2613	7.57E-11	30	117	0.7571	0	20	59	0.7026	6.44E-11	27	75	0.7996	3.24E-11
	5 × 10^4^	$x_{0}^{3}$	14	28	0.2852	2.48E-11	20	38	0.3575	5.11E-11	19	44	0.5344	5.19E-11	28	67	0.7826	8.50E-11
	5 × 10^4^	$x_{0}^{4}$	13	26	0.2589	2.58E-11	2	3	0.0426	0	19	46	0.5896	3.11E-11	27	76	0.8396	7.57E-11
	5 × 10^4^	$x_{0}^{5}$	14	28	0.2909	3.88E-11	4	5	0.0758	0	***	***	***	***	25	75	0.7601	7.36E-11
	5 × 10^4^	$x_{0}^{6}$	13	26	0.2661	3.16E-11	2	3	0.0486	0	19	46	0.5384	4.57E-11	27	73	0.7991	8.92E-11

**Table 2:** SDYM against SCGP, SRMIL, and MDY numerical results for examples 3 and 4.

PN	VAR	IG	SDYM				SCGP				MDY				SRMIL			
			NOI	FV	Ptime	Norm	NOI	FV	Ptime	Norm	NOI	FV	Ptime	Norm	NOI	FV	Ptime	Norm
3	10^3^	$x_{0}^{1}$	10	18	0.0101	1.78E-11	12	22	0.0100	9.71E-11	14	38	0.0308	6.82E-11	267	386	0.2454	9.79E-11
	10^3^	$x_{0}^{2}$	10	18	0.0109	1.78E-11	12	22	0.0102	9.71E-11	14	38	0.0220	6.82E-11	257	377	0.2359	9.52E-11
	10^3^	$x_{0}^{3}$	10	18	0.0105	1.78E-11	12	22	0.0095	9.71E-11	14	38	0.0258	6.82E-11	231	342	0.2112	9.53E-11
	10^3^	$x_{0}^{4}$	10	18	0.0122	1.78E-11	12	22	0.0097	9.71E-11	14	38	0.0259	6.82E-11	239	351	0.2304	9.72E-11
	10^3^	$x_{0}^{5}$	10	18	0.0112	1.78E-11	12	22	0.0099	9.71E-11	14	38	0.0335	6.82E-11	257	377	0.2229	9.52E-11
	10^3^	$x_{0}^{6}$	10	18	0.0127	1.78E-11	12	22	0.0172	9.71E-11	14	38	0.0269	6.82E-11	131	207	0.1408	9.67E-11
	10^4^	$x_{0}^{1}$	16	33	0.0909	5.38E-11	13	38	0.0814	6.15E-11	15	36	0.1382	1.53E-11	20	82	0.2007	4.10E-11
	10^4^	$x_{0}^{2}$	16	33	0.0934	5.38E-11	13	38	0.0839	6.15E-11	15	36	0.1398	1.53E-11	21	86	0.2173	4.38E-11
	10^4^	$x_{0}^{3}$	16	33	0.0906	5.38E-11	13	38	0.0765	6.15E-11	15	36	0.1542	1.53E-11	21	84	0.2141	3.85E-11
	10^4^	$x_{0}^{4}$	16	33	0.1001	5.38E-11	13	38	0.0798	6.15E-11	15	36	0.1474	1.53E-11	22	82	0.2133	6.75E-11
	10^4^	$x_{0}^{5}$	16	33	0.0900	5.38E-11	13	38	0.0785	6.14E-11	15	36	0.1229	1.53E-11	21	86	0.1944	4.37E-11
	10^4^	$x_{0}^{6}$	16	33	0.0911	5.38E-11	13	38	0.0805	6.14E-11	15	36	0.1291	1.53E-11	28	110	0.2634	4.28E-11
	5 × 10^4^	$x_{0}^{1}$	12	37	0.3158	6.64E-11	13	48	0.3242	6.54E-11	14	58	0.5360	6.50E-11	17	99	0.7366	6.57E-11
	5 × 10^4^	$x_{0}^{2}$	12	37	0.2853	6.64E-11	13	48	0.3011	6.58E-11	16	58	0.5825	2.32E-11	18	108	0.7765	9.51E-11
	5 × 10^4^	$x_{0}^{3}$	12	37	0.2986	6.64E-11	13	48	0.3344	6.58E-11	15	56	0.5357	1.07E-11	20	118	0.8683	1.36E-11
	5 × 10^4^	$x_{0}^{4}$	12	37	0.3242	6.64E-11	13	48	0.3147	6.58E-11	18	85	0.7478	5.01E-11	19	110	0.8442	4.96E-11
	5 × 10^4^	$x_{0}^{5}$	12	37	0.2998	6.64E-11	13	48	0.3219	6.58E-11	16	58	0.5727	1.15E-11	18	108	0.7626	9.51E-11
	5 × 10^4^	$x_{0}^{6}$	12	37	0.2941	6.64E-11	13	48	0.3862	6.58E-11	16	55	0.5615	4.50E-11	19	114	0.8599	1.96E-11
4	10^3^	$x_{0}^{1}$	9	28	0.0126	6.02E-11	31	200	0.0309	7.75E-11	20	76	0.0364	2.35E-11	24	121	0.0299	5.71E-11
	10^3^	$x_{0}^{2}$	11	33	0.0129	1.46E-11	21	133	0.0232	4.45E-11	18	55	0.0298	5.18E-11	27	132	0.0325	1.82E-11
	10^3^	$x_{0}^{3}$	11	33	0.0097	5.98E-11	9	30	0.0119	8.49E-11	19	69	0.0477	6.56E-11	24	120	0.0468	8.64E-11
	10^3^	$x_{0}^{4}$	11	33	0.0096	1.97E-11	20	88	0.0173	6.13E-11	21	100	0.0429	8.76E-11	28	138	0.0618	3.23E-11
	10^3^	$x_{0}^{5}$	13	41	0.0162	8.19E-11	22	111	0.0205	8.82E-11	16	62	0.0410	9.50E-11	30	146	0.0718	5.08E-11
	10^3^	$x_{0}^{6}$	13	38	0.0121	3.22E-11	10	34	0.0115	1.01E-11	16	58	0.0342	6.82E-11	31	147	0.0558	3.57E-11
	10^4^	$x_{0}^{1}$	9	31	0.0675	8.78E-11	43	335	0.3610	1.26E-11	16	54	0.1660	7.41E-11	24	122	0.2156	9.60E-11
	10^4^	$x_{0}^{2}$	11	33	0.0735	4.61E-11	35	276	0.2552	5.19E-11	19	63	0.1903	7.31E-11	27	132	0.2378	5.76E-11
	10^4^	$x_{0}^{3}$	12	36	0.0672	2.98E-11	10	30	0.0613	4.58E-11	19	70	0.1984	2.11E-11	27	135	0.2291	6.09E-11
	10^4^	$x_{0}^{4}$	11	33	0.0654	6.22E-11	21	102	0.1340	6.35E-11	23	107	0.2506	3.22E-11	29	143	0.2746	4.52E-11
	10^4^	$x_{0}^{5}$	14	41	0.0766	7.35E-11	29	155	0.2026	5.57E-11	23	92	0.2318	8.75E-11	32	156	0.2877	5.79E-11
	10^4^	$x_{0}^{6}$	13	41	0.0760	4.71E-11	11	34	0.0680	2.68E-11	19	68	0.1924	3.18E-11	32	151	0.3024	3.42E-11
	5 × 10^4^	$x_{0}^{1}$	10	31	0.2110	8.68E-12	32	191	0.8711	7.66E-11	19	68	0.5870	3.81E-11	25	127	0.9004	4.55E-11
	5 × 10^4^	$x_{0}^{2}$	11	36	0.2509	5.73E-11	24	160	0.7099	2.64E-13	18	56	0.5549	5.72E-11	29	142	1.0063	6.15E-11
	5 × 10^4^	$x_{0}^{3}$	12	36	0.2689	6.67E-11	10	33	0.2228	3.06E-11	19	69	0.6315	3.79E-11	28	140	0.9493	1.23E-11
	5 × 10^4^	$x_{0}^{4}$	11	36	0.2598	7.75E-11	21	91	0.4956	2.94E-12	21	99	0.8176	5.22E-11	30	148	1.0289	1.23E-11
	5 × 10^4^	$x_{0}^{5}$	14	44	0.3139	7.60E-11	28	148	0.7364	2.42E-11	21	86	0.7727	6.90E-11	33	161	1.0975	5.79E-11
	5 × 10^4^	$x_{0}^{6}$	14	41	0.2863	4.65E-12	14	67	0.3468	1.89E-12	20	72	0.6750	4.64E-11	32	151	1.1119	7.65E-11

**Table 3:** SDYM against SCGP, SRMIL, and MDY numerical results for examples 5 and 6.

PN	VAR	IG	SDYM				SCGP				MDY				SRMIL			
			NOI	FV	Ptime	Norm	NOI	FV	Ptime	Norm	NOI	FV	Ptime	Norm	NOI	FV	Ptime	Norm
5	10^3^	$x_{0}^{1}$	41	123	0.0352	9.86E-11	***	***	***	***	99	537	0.1597	6.25E-11	85	442	0.1369	8.52E-11
	10^3^	$x_{0}^{2}$	44	134	0.0425	8.97E-11	***	***	***	***	60	296	0.1140	8.24E-11	266	1277	0.3183	8.87E-11
	10^3^	$x_{0}^{3}$	36	110	0.0361	3.52E-11	***	***	***	***	129	687	0.2189	7.19E-11	219	1079	0.3053	9.81E-11
	10^3^	$x_{0}^{4}$	39	118	0.0385	9.33E-11	***	***	***	***	172	957	0.2329	8.48E-11	227	1116	0.2955	9.03E-11
	10^3^	$x_{0}^{5}$	53	161	0.0527	8.22E-11	***	***	***	***	188	1108	0.3700	8.00E-11	251	1224	0.3177	8.90E-11
	10^3^	$x_{0}^{6}$	47	142	0.0398	6.31E-11	***	***	***	***	115	669	0.1895	7.81E-11	259	1249	0.3264	8.82E-11
	10^4^	$x_{0}^{1}$	39	120	0.2130	4.29E-11	***	***	***	***	62	321	0.5744	2.98E-11	82	430	0.7386	8.17E-11
	10^4^	$x_{0}^{2}$	58	177	0.3025	8.13E-11	***	***	***	***	67	341	0.5474	6.62E-11	266	1286	2.0663	9.82E-11
	10^4^	$x_{0}^{3}$	44	134	0.2384	6.97E-11	***	***	***	***	180	1034	1.4526	9.55E-11	242	1183	1.8472	9.14E-11
	10^4^	$x_{0}^{4}$	46	138	0.2370	6.75E-11	***	***	***	***	107	621	0.9360	7.53E-11	237	1166	1.9006	9.48E-11
	10^4^	$x_{0}^{5}$	58	176	0.3042	9.09E-11	***	***	***	***	163	905	1.2799	8.34E-11	272	1317	2.0601	8.70E-11
	10^4^	$x_{0}^{6}$	51	157	0.3090	8.81E-11	***	***	***	***	112	610	0.8982	9.09E-11	276	1326	2.0889	8.90E-11
	5 × 10^4^	$x_{0}^{1}$	42	129	0.9898	7.35E-11	***	***	***	***	30	132	1.1826	9.88E-11	131	664	5.1011	9.70E-11
	5 × 10^4^	$x_{0}^{2}$	53	157	1.2067	7.47E-11	***	***	***	***	124	682	5.0419	6.25E-11	259	1265	9.3065	8.41E-11
	5 × 10^4^	$x_{0}^{3}$	44	131	1.0058	8.23E-11	***	***	***	***	148	821	5.9841	6.98E-11	261	1268	9.3409	9.83E-11
	5 × 10^4^	$x_{0}^{4}$	44	136	1.0487	8.50E-11	***	***	***	***	185	1042	7.5030	6.23E-11	274	1323	9.7754	8.77E-11
	5 × 10^4^	$x_{0}^{5}$	51	150	1.1779	7.36E-11	***	***	***	***	160	888	6.4943	6.68E-11	275	1336	9.7507	9.47E-11
	5 × 10^4^	$x_{0}^{6}$	52	159	1.2986	7.81E-11	***	***	***	***	163	885	6.5324	7.30E-11	277	1336	9.8300	9.71E-11
6	10^3^	$x_{0}^{1}$	44	156	0.0630	5.74E-11	***	***	***	***	45	240	0.1098	4.91E-11	25	183	0.0696	7.06E-11
	10^3^	$x_{0}^{2}$	58	201	0.0575	8.37E-11	***	***	***	***	39	172	0.0565	7.14E-11	28	201	0.0697	3.68E-11
	10^3^	$x_{0}^{3}$	45	157	0.0745	5.37E-11	***	***	***	***	54	271	0.0985	5.86E-11	28	200	0.0482	8.03E-11
	10^3^	$x_{0}^{4}$	46	160	0.0649	7.67E-11	***	***	***	***	38	158	0.0928	3.75E-11	28	202	0.0641	7.11E-11
	10^3^	$x_{0}^{5}$	48	167	0.0550	8.61E-11	***	***	***	***	43	195	0.0943	5.06E-11	28	201	0.0666	4.64E-11
	10^3^	$x_{0}^{6}$	56	191	0.0688	7.73E-11	***	***	***	***	42	190	0.0999	9.35E-11	27	195	0.1058	7.04E-11
	10^4^	$x_{0}^{1}$	47	166	0.2960	7.24E-11	***	***	***	***	55	293	0.5936	6.87E-11	26	189	0.3766	8.77E-11
	10^4^	$x_{0}^{2}$	59	205	0.3485	9.24E-11	***	***	***	***	37	152	0.3791	7.59E-11	28	201	0.4016	6.54E-11
	10^4^	$x_{0}^{3}$	49	172	0.2883	7.42E-11	***	***	***	***	47	218	0.5567	9.50E-11	55	364	0.6229	4.08E-11
	10^4^	$x_{0}^{4}$	46	162	0.2890	7.25E-11	***	***	***	***	38	148	0.3734	7.68E-11	28	202	0.3624	7.43E-11
	10^4^	$x_{0}^{5}$	52	178	0.3234	8.72E-11	***	***	***	***	44	194	0.4180	7.84E-11	29	208	0.4063	3.03E-11
	10^4^	$x_{0}^{6}$	57	197	0.3547	6.47E-11	***	***	***	***	44	180	0.4390	4.48E-11	28	202	0.3858	8.76E-11
	5 × 10^4^	$x_{0}^{1}$	50	174	1.2282	8.33E-11	***	***	***	***	52	258	2.2164	3.84E-11	27	196	1.4190	4.32E-11
	5 × 10^4^	$x_{0}^{2}$	61	212	1.5210	6.83E-11	***	***	***	***	49	224	2.0231	6.13E-11	29	208	1.5456	3.76E-11
	5 × 10^4^	$x_{0}^{3}$	56	190	1.4193	7.25E-11	***	***	***	***	46	218	1.8750	8.11E-11	28	202	1.4516	9.27E-11
	5 × 10^4^	$x_{0}^{4}$	57	196	1.3920	7.81E-11	***	***	***	***	39	150	1.4818	4.45E-11	29	209	1.5536	7.72E-11
	5 × 10^4^	$x_{0}^{5}$	53	184	1.3149	5.21E-11	***	***	***	***	48	214	1.8956	6.15E-11	29	208	1.5170	6.15E-11
	5 × 10^4^	$x_{0}^{6}$	59	204	1.4770	9.57E-11	***	***	***	***	51	232	2.0667	9.64E-11	29	209	1.5535	4.95E-11

**Fig 1:**
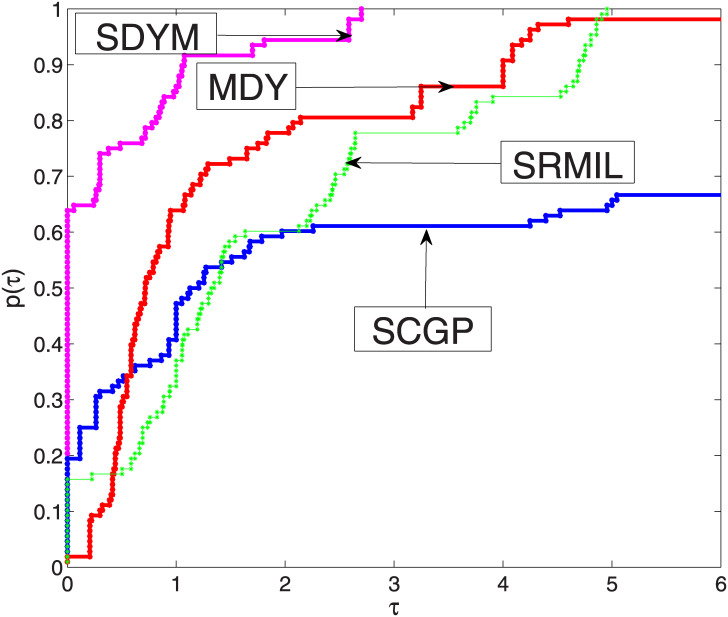
Performance of SDYM against SCGP, SRMIL, and MDY for iterations.

**Fig 2:**
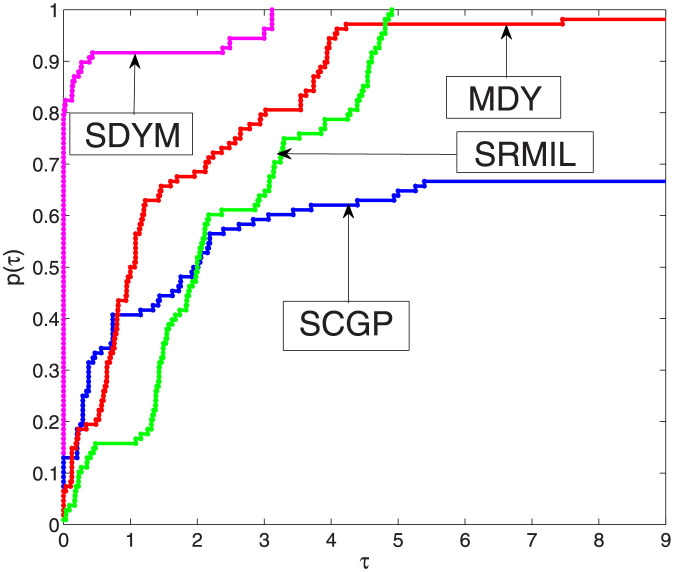
Performance SDYM against SCGP, SRMIL, and MDY for function evaluations.

**Fig 3:**
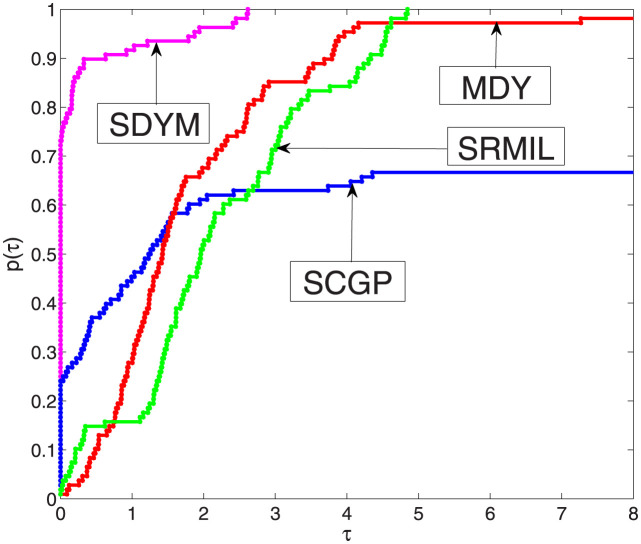
Performance SDYM against SCGP, SRMIL, and MDY for CPU time.

From Tables 1–3 we observe that SDYM algorithms are more effective compared to the other three algorithms since they successfully solved all six examples. To further explain the performance of each algorithm, the performance tool developed in [62] by Dolan and Moré to plot Figs 1–3, with respect to our metrics performance, namely; function values, iterations, and processing time for each algorithm. The *y*-axis of each figure represents the examples solved with a minimum value of the aforementioned metrics; the right side of each figure represents the percentage of the examples solved by each algorithm successfully, while the algorithms that maintain the topmost curve, indicate the scheme that solves the majority of considered examples within a factor *τ* of the best time. Fig 1 indicates that the SDYM algorithm solved 62.96% of the test examples with minimum iterations, while SCGP, MDY and SRMIL recorded 19.45%, 1.85% and 15.74% respectively. As exhibited by Fig 2, the SDYM algorithm solved 79.63% of the test examples with fewer function values, while SCGP, MDY and SRMIL recorded 12.96%, 6.48% and 0.93% respectively. Regarding minimum CPU time metric, Fig 3 showed that SDYM is the fastest since it solved 73.15% of the test examples compared to SCGP, MDY and SRMIL algorithms recorded 24.07%, 0.93% and 1.85% respectively. In addition to the above analysis, the curve representing the SDYM algorithm remains at the top of the curves representing the SCGP, MDY and SRMIL algorithms. Based on this discussion, it can be concluded that the SDYM algorithm is promising for solving (14).

## Sparse signal reconstruction

Here, we discuss the application of the SDYM algorithm in solving signal reconstruction problems. To that end and to further test its performance, the SDYM algorithm is compared with HTTCGP [40] and PCG [38] algorithms. Sparse signal reconstruction refers to a technique of reconstructing a disturbed signal in a sparse scenario. This procedure comes up often in practical disciplines, which include astrophysical signals, machine learning, wireless sensor networks, video coding, medical imaging, radar, and compressive imaging [63]. The target of the technique is obtaining sparse solutions to the under-determined linear system Sx=b, in which $S\in R^{k\times n}\left(k\ll n\right)$ is a sampling matrix, *x* denotes the original sparse signal and $b\in R^{k}$ stands for an observed value. Hence, in order to reconstruct *x* from the system Sx=b, the *ℓ*_1_*norm* regularization problem

$$\begin{array}{c} \underset{x}{\text{min}\;}f\left(x\right):=\frac{1}{2}{∥b-Sx∥}_{2}^{2}+\gamma {∥x∥}_{1}, \end{array}$$ (49)

is solved where *γ* > 0 is a parameter.

In solving (49), the authors in [64] reformulated it as a convex quadratic model by first splitting a vector $x\in R^{n}$ into two parts, i.e.,

$$\begin{array}{c} x=v-w,\;v\geq 0,\;w\geq 0,\;v,w\in R^{n}, \end{array}$$

where *v*_*i*_ = (*x*_*i*_)_+_, *w*_*i*_ = (−*x*_*i*_)_+_, ∀*i* = 1, 2, …, *n* and (.)_+_ = max{0, *x*}. By this formulation, we can write ${∥x∥}_{1}=E_{n}^{T}v+E_{n}^{T}w$ where $E_{n}={\left(1,1,\ldots ,1\right)}^{T}\in R^{n}$. Consequently, (49) can be re-formulated as

$$\begin{array}{c} \text{min}\;\{\frac{1}{2}{∥S\left(v-w\right)-b∥}_{2}^{2}+\gamma \left(E_{n}^{T}v+E_{n}^{T}w\right)|v\geq 0,w\geq 0\}. \end{array}$$ (50)

By setting the variables:

$$\begin{array}{c} z=(\begin{array}{c} v\\ w \end{array}),\;\chi =\gamma E_{2n}+(\begin{array}{c} -\omega \\ \omega \end{array}),\;\omega =S^{T}b,\;H=(\begin{array}{cc} S^{T}S & -S^{T}S\\ -S^{T}S & S^{T}S \end{array}), \end{array}$$

(50) can be reformulated as

$$\begin{array}{c} \text{min}\;\{\frac{1}{2}z^{T}Hz+\chi^{T}z|\;z\geq 0\}, \end{array}$$ (51)

as a standard bound-constrained quadratic programming problem. This problem was restarted as

$$\begin{array}{c} F(z)=\text{min}\;\{z,Hz+\chi \}=0. \end{array}$$

which clearly represents the convex quadratic programming problem, see [65]. Since it was proven in [65, 66] that *F* is monotone and Lipschitz continuous, then (49) can equivalently be represented as (14), which can be solved with the SDYM algorithm.

### Experiments and reported results

Here, the SDYM algorithm is applied from some *k* observations to reconstruct a sparse signal of length *n* with *k* ≪ *n*. For the experiments, the HTTCGP and PCG algorithms were employed to compare the performances of SDYM since they are used to solve signal problems. The PCG and HTTCGP algorithms were run with the same values as used in the original papers. Apart from *ζ* = 0.4, the SDYM parameter values stay the same as in the previous experiment. The quality of restoration is tested in the experiments by the mean square error (MSE), which is defined by

$$\begin{array}{c} MSE=\frac{1}{n}{∥\hat{x}-\bar{x}∥}^{2}, \end{array}$$

where $\hat{x}$ is the reconstructed signal and $\bar{x}$ is the actual one. This definition shows that a scheme with lower MSE yields better-quality reconstructed signals. We used *n* = 2^12^ as the size of the signal with *k* = 2^10^, and 2^6^ as the randomly nonzero elements contained in the original signal. In Matlab, the Gaussian matrix S is produced using the function randn(k, n). Furthermore, noise, particularly

$$\begin{array}{c} b=S\hat{x}-\eta \end{array}$$

interferes with the measurement *b*, where *η* is the Gaussian noise distributed as *N*(0, 10^−4^). To initiate the experiment, we used the measurement image, i.e., $x_{0}=S^{T}b$, which terminates if the inequality

$$\begin{array}{c} \frac{∥f_{k}-f_{k-1}∥}{∥f_{k-1}∥}<10^{-5}, \end{array}$$

holds, where the function $f_{k}=\frac{1}{2}∥Sx_{k}{-b∥}_{2}^{2}+\gamma {∥x_{k}∥}_{1}$, with the parameter *γ* > 0.

**Fig 4:**
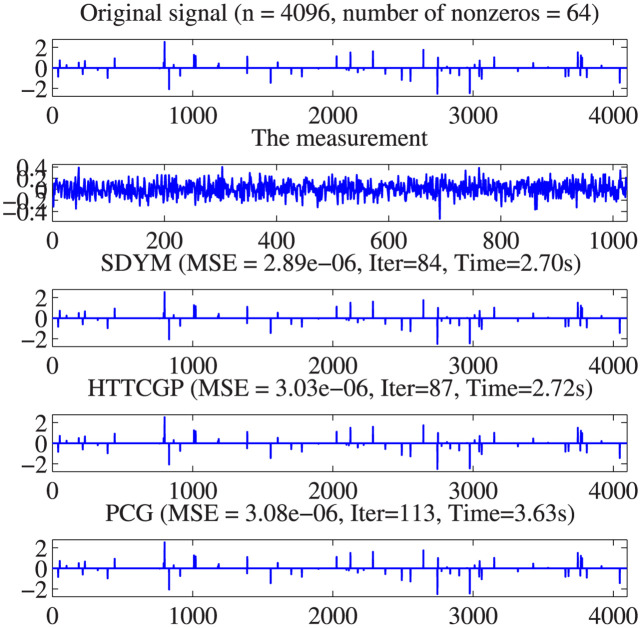
Original, measurement and restored signals comparison for the SDYM, HTTCGP, and PCG algorithms.

**Fig 5:**
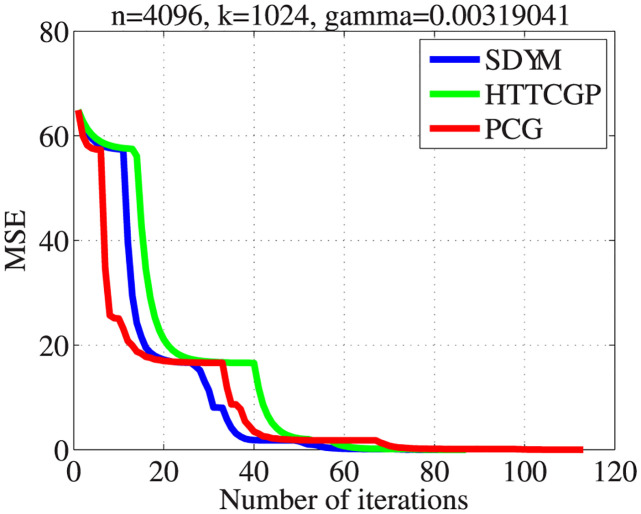
The comparison for the number of MSE against the number of iterations.

**Fig 6:**
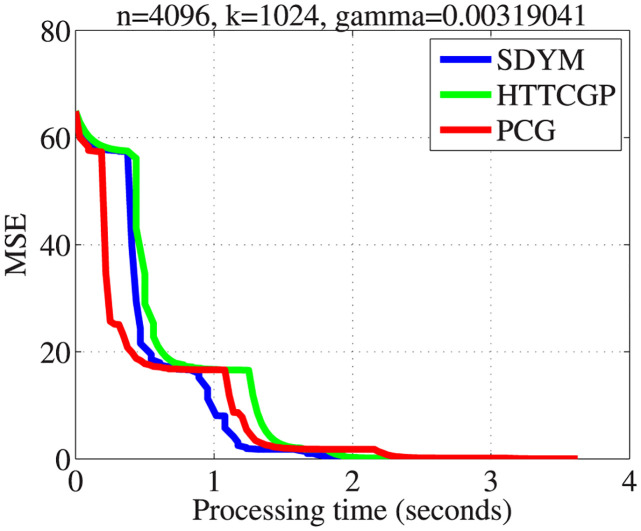
The comparison for the number of MSE against processing time (seconds).

**Fig 7:**
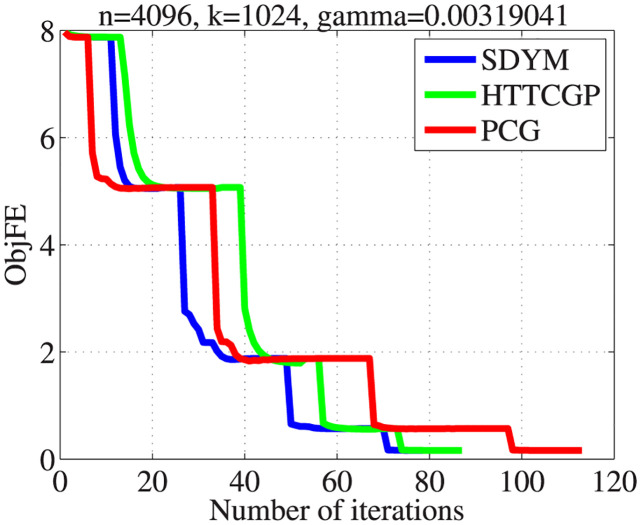
The comparison for the number of the objective function against the number of iterations.

**Fig 8:**
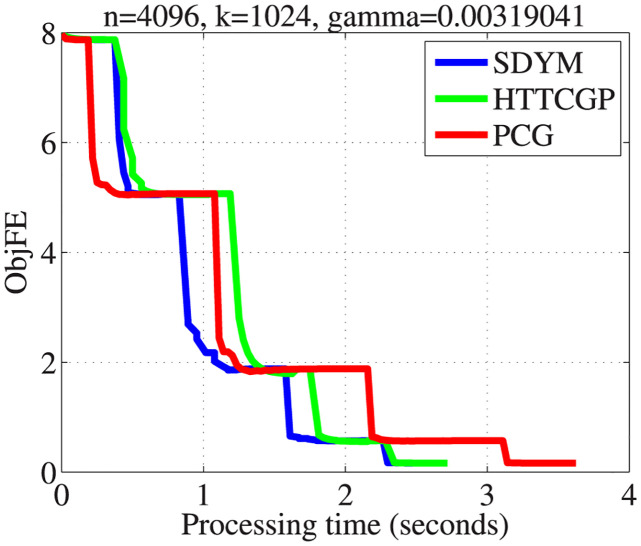
The comparison for the number of the objective function against processing time (seconds).

The actual sparse signal, its measurement, and reconstructed version by the three algorithms are displayed in Fig 4. It can be observed from plots 5–8 in descending order that the three algorithms were able to reconstruct the signal almost exactly from the measurement, with SDYM achieving that much faster. In addition, Figs 5–8 consist of four graphs that depict the convergence performance of the three methods in terms of mean square error (MSE), function values (ObjFE), processing time and number of iterations metrics. Thus, as observed from Figs 5–8, the rates of descent of MSE and ObjFE for SDYM are much faster than HTTCGP and PCG methods. In addition, the experiment was repeated times, and the results are given in Table 4. The results displayed HTTCGP and PCG in three of the four metrics considered. These results clearly showed that the SDYM algorithm is effective for decoding sparse signals in CP.

**Table 4:** The signal reconstruction report of twelve noisy experiments.

SDYM					HTTCGP				PCG			
Exp. No	MSE	NOI	ObjFE	PTime	MSE	NOI	ObjFE	PTime	MSE	NOI	ObjFE	PTime
1	2.885e-06	84	1.637e-01	2.70	3.031e-06	87	1.636e-01	2.72	3.076e-06	113	1.636e-01	3.63
2	8.889e-06	75	2.917e-01	2.55	8.930e-06	82	2.916e-01	2.66	9.320e-06	99	2.915e-01	3.44
3	2.867e-06	79	1.874e-01	2.67	3.001e-06	87	1.873e-01	2.91	3.095e-06	102	1.873e-01	3.45
4	2.908e-06	83	1.824e-01	2.80	2.918e-06	88	1.824e-01	2.98	3.113e-06	101	1.823e-01	3.36
5	2.148e-06	80	1.414e-01	2.55	2.174e-06	83	1.414e-01	2.72	2.312e-06	107	1.413e-01	3.45
6	2.869e-06	87	1.633e-01	2.91	2.934e-06	88	1.632e-01	2.84	2.977e-06	110	1.632e-01	3.55
7	3.673e-06	86	1.825e-01	2.75	3.695e-06	94	1.824e-01	3.16	3.844e-06	109	1.824e-01	3.72
8	3.428e-06	84	2.145e-01	2.92	3.416e-06	86	2.145e-01	2.92	3.658e-06	106	2.144e-01	3.67
9	2.484e-06	77	1.705e-01	2.58	2.472e-06	82	1.705e-01	3.19	2.606e-06	102	1.704e-01	3.39
10	2.267e-06	73	1.335e-01	2.13	2.352e-06	84	1.335e-01	2.55	2.377e-06	103	1.335e-01	3.16
11	2.279e-06	75	1.123e-01	2.41	1.796e-06	84	1.126e-01	2.69	1.930e-06	93	1.125e-01	3.08
12	2.258e-06	80	8.523e-02	2.67	1.354e-06	91	8.535e-02	2.94	1.434e-06	107	8.534e-02	3.47
Average	3.246e-06	80.25	2.330e-02	2.64	3.807e-06	86.33	2.330e-02	2.86	3.974e-06	104.33	2.330e-02	3.45

## Conclusion

In this paper, we presented a spectral-type Dai-Yuan algorithm for a constrained NCMS and reconstructed sparse signals in CP. The new algorithm fulfils the sufficient DC irrespective of the line search technique applied. The proposed algorithm’s global convergence was shown using a monotone line search approach and certain fundamental assumptions. Furthermore, a comparison of the proposed approach to three efficient methods shows that it is efficient.

## Supporting information

S1 Data (BST)
